# Honors for Dr. Jacobi

**Published:** 1900-05

**Authors:** 


### HONORS FOR DR. JACOBI.

DR. ABRAHAM JACOBI, of New York will attain the 70th year of his age May 6, 1900. It is proposed by a number of his friends and professional associates to tender him a subscription dinner in commemoration of the event.

It is a remarkable coincidence that Dr. Jacobi should reach almost simultaneously the 30th year of his teaching as clinical professor of diseases of children at the College of Physicians and Surgeons. The class of 1900 at the college appropriately celebrated these events April 25, 1900, when Dr. Jacobi delivered the last lecture for the year in his course. The students gathered in the big theatre of Vanderbilt Clinic to give their professor a loving cup filled with flowers tied with the college colors, blue and white, and framed resolutions signed by the members of the class, telling of their esteem and of their hope for his prolonged activity in medicine.

R. T. Frank, '00, presided, and E. L. Trudeau, of the same class, made the presentation. Dr. Jacobi thanked them, and said that the secret of his appearance before them was that he always worked with younger men. This he was sure, constituted a preventive to old age.

The dinner above referred to will be given at Delmonico's, 44th Street and 5th Avenue, New York, Saturday evening, May 5th. The subscription price of the dinner will be $10.00, including wine, and those desiring to attend should notify Dr. A. G. Gerster, chairman of committee, P. O. Box 3032, New York.

It is announced that Dr. Joseph D. Bryant will preside and that Carl Schurz, Seth Low, William Dean Howells, Professor William H. Thomson and William Osler, of Baltimore, will speak.

The principal event of the evening, according to further announcement, will be the presentation to Dr. Jacobi of a "festschrift," a way of honoring a prominent man up to this time never used in this country. Famous Americans, Germans, Englishmen, Frenchmen, Spaniards and Austrians, most of them specialists, have written for the book. Dr. Bryant will make the presentation speech. Many associations will be represented, and the German Medical Society has arranged to give Professor Jacobi a set of resolutions and an engrossed album containing the members names.

Dr. Jacobi was born in Hartum, Westphalia, on May 6, 1830. He studied in the universities of Greifswald, Göttingen and Bonn. At the last place he received the doctorate degree. When the revolu tion broke out, although only a boy of eighteen, he joined the Young German party and enlisted in the army.

At Berlin in 1849 he was arrested, together with Marx, Freiligrath and Becker. He was tried for treason, and taken to Cologne and thrown into prison, where he remained for eighteen months. When, after much cruel treatment, he was released he was tried and acquitted, but immediately after was rearrested and sentenced to six months in Bielfeld Castle for an alleged insult to the Crown.

In 1853 Dr. Jacobi came to New York, and became lecturer in infantile pathology in the College of Physicians and Surgeons, and in 1860 he took the first chair of children's diseases established in America, that in the New York Medical College. In 1870 he became clinical professor of diseases of children in the College of Physicians and Surgeons. In 1898 the University of Michigan bestowed on him the degree of LL.D. He has served in many New York Hospitals, has been an officer in the prominent medical societies, written several well known works, and has been made honorary president of the International Medical Congress of 1900 in Paris, where he will read a paper on Infant Feeding.

---

THE tenement-house commission recently appointed by Governor Roosevelt, is constituted as follows: Messrs. Raymond T. Almirall, of Brooklyn; Hugh Bonner, Paul D. Cravath, Robert W. DeForest, of Manhattan; William A. Douglas, of Buffalo; Otto M. Eidlitz, of Manhattan; F. Norton Goddard, of Manhattan; William Lansing, of Buffalo; William J. O'Brien, of Manhattan; James B. Reynolds, of Manhattan; I. N. Phelps Stokes, of Manhattan; Myles Tierney, of Manhattan; Alfred T. White of Brooklyn and Dr. George B. Fowler, of Manhattan. The duty of the commission is to make careful examination into the tenement houses in New York and Buffalo, their condition as to the construction, healthfulness, safety, rentals, and the effect of tenement-house life on the health, education, savings, and morals of those who live in tenement houses, and all other phases of the so-called tenement-house question in these cities that can affect the public welfare. An appropriation of $10,000 is made for the expenses of the commission, which may subpena and compel the attendance of witnesses, employ counsel, assistants, and experts, and make such recommendations to the next legislature as it deems wise, to enable the best and highest possible condition for tenement-house life in New York and Buffalo to be attained.—*Med. Record.*

---

As indicating whether antitoxin for diphtheria has done anything for Buffalo or not the following table which is a record of all cases of the disease reported and the number of deaths from the disease during the years mentioned, will show :

DEATH-RATE FROM DIPHTHERIA.

| Year. | Cases Reported. | Deaths. | Percentage of Deaths. |
|---|---|---|---|
| 1893[1] | 455 | 149 | 33 |
| 1894 | 534 | 214 | 40 |
| 1895[2] | 851 | 242 | 28.5 |
| 1896 | 1,071 | 249 | 23 |
| 1897 | 1,168 | 198 | 17 |
| 1898 | 622 | 85 | 13 |
| 1899 | 609 | 83 | 13 |

---

The Secretary of the Treasury has submitted to the House a request for $200,000 additional to the fund to prevent the introduction and spread of epidemic diseases. He says the Surgeon-General of the Marine Hospital Service reports that, on account of the continued and increasing danger from plague, medical officers have been stationed at United States consulates in Europe from which emigrants depart. Medical officers have also been stationed at the fruit ports

1. Inauguration of bureau of bacteriology.

2. The beginning of bacteriological diagnosis *almost universally* by physicians. The increases during the following years of reported cases due to the fact that many illnesses formerly called "tonsillitis" were found to be diphtheritic in origin.

of Central and South America to guard against yellow fever. The consulates at Yokohama, Kobe and Hong Kong also have medical officers. He continues as follows:

The Surgeon-General further states that plague has recently been reported at San Francisco, and that the early recrudescence of yellow fever in Florida and other southern states is seriously apprehended.

To meet this state of affairs the Secretary asks for $200,000 in addition to the $300,000 heretofore given, and he requests that it be made immediately available, "since the appropriation is almost exhausted, and should yellow fever appear during the month of June a deficit would be unavoidable."

This is a timely and wise suggestion on the part of Secretary Gage, and congress should make speedy response to the request. There should be neither delay nor parsimony in guarding against epidemic disease.

---

TRAINED nurses are wanted in Cuba, according to Assistant Adjutant-General St. J. Greble, Superintendent of hospitals and charities at Havana, who has written to the Bellevue Hospital authorities at New York, that he desires to place a trained nurse in each hospital and orphan asylum in Cuba that has more than 100 inmates, "to straighten out the present deplorable condition of most of these institutions." He asks the authorities to recommend men and women who would be willing to undertake the work.

---

Two acts have been passed by the state legislature providing hospitals for New York City. One establishes a hospital for the treatment of contagious eye diseases, food, medicine and supplies to be furnished by the health department; the other provides for an appropriation of $350,000 for site and erection of a hospital for pulmonary tuberculosis, the New York City Board of Health to have jurisdiction, and prescribe rules and regulations for its government.

---

MAJOR TAYLOR, the United States Army Surgeon, in charge of the military hospital at Honolulu reports to the Surgeon-General that the ravages of the plague are at an end. The medical officers have been hard pressed and overworked in their battle with the plague at Honolulu, but they have kept conscientiously at it and have won a brilliant victory.

